# Mixotrophic Cultivation of a Native Cyanobacterium, *Pseudanabaena mucicola* GO0704, to Produce Phycobiliprotein and Biodiesel

**DOI:** 10.4014/jmb.2207.07008

**Published:** 2022-09-09

**Authors:** Shin Myung Kim, Eun Hee Bae, Jee Young Kim, Jae-Shin Kang, Yoon-E Choi

**Affiliations:** 1 Division of Environmental Science and Ecological Engineering, Korea University, Seoul 02841, Republic of Korea; 2 Research Division of Microorganisms, National Institute of Biological Resources, Incheon 22689, Republic of Korea; 3 Research Division of Plants, National Institute of Biological Resources, Incheon, Republic of Korea (present address)

**Keywords:** Biofuel, cyanobacteria, phycobiliprotein, organic carbon sources, biodiesel quality

## Abstract

Global warming has accelerated in recent decades due to the continuous consumption of petroleum-based fuels. Cyanobacteria-derived biofuels are a promising carbon-neutral alternative to fossil fuels that may help achieve a cleaner environment. Here, we propose an effective strategy based on the large-scale cultivation of a newly isolated cyanobacterial strain to produce phycobiliprotein and biodiesel, thus demonstrating the potential commercial applicability of the isolated microalgal strain. A native cyanobacterium was isolated from Goryeong, Korea, and identified as *Pseudanabaena mucicola* GO0704 through 16s RNA analysis. The potential exploitation of *P. mucicola* GO0704 was explored by analyzing several parameters for mixotrophic culture, and optimal growth was achieved through the addition of sodium acetate (1 g/l) to the BG-11 medium. Next, the cultures were scaled up to a stirred-tank bioreactor in mixotrophic conditions to maximize the productivity of biomass and metabolites. The biomass, phycobiliprotein, and fatty acids concentrations in sodium acetate-treated cells were enhanced, and the highest biodiesel productivity (8.1 mg/l/d) was achieved at 96 h. Finally, the properties of the fuel derived from *P. mucicola* GO0704 were estimated with converted biodiesels according to the composition of fatty acids. Most of the characteristics of the final product, except for the cloud point, were compliant with international biodiesel standards [ASTM 6761 (US) and EN 14214 (Europe)].

## Introduction

Industrialization, lifestyle modernization, and significant increases in the number of automobiles have greatly increased the demand for petroleum-based fuels. Currently, approximately 85% of the planet’s primary energy requirement is met by petroleum-based fuels [1]. However, steady increases in fossil fuel consumption have resulted in global warming because of the build-up of carbon dioxide in the air [2]. This continuous rise in global temperature may wipe out 39%–43% of the world’s biota [3]. In the short term, other associated problems may occur, including air quality deterioration, changes in disease patterns, and reduced food supplies [4]. Therefore, the partial or complete replacement of fossil fuels with renewable clean energy is urgently needed to ensure global stability and human affairs.

Biofuels derived from photosynthetic microorganisms are an attractive alternative to petroleum fuels due to (1) the rapid growth rate, (2) space-efficient cultivation, (3) high lipid accumulation ability, and (4) high carbon fixation rate of microalgae [1, 5, 6]. Photosynthetic microorganisms are capable of capturing carbon dioxide and converting solar energy into chemical energy, thus providing an alternative for the production of sustainable and carbon-neutral energy sources [5, 7]. Cyanobacteria, a gram-negative prokaryotic autotroph, are microbial organisms ubiquitously found in natural waters that play a pivotal role in biogeochemical cycles [8]. Furthermore, in addition to photosynthesis, cyanobacteria produce important commercial pigments (astaxanthin, lutein, phycobiliprotein), vitamins, and essential nutrients (notably proteins, carbohydrates, and lipids) [9]. However, many cyanobacteria also produce a variety of detrimental toxic substances known as cyanotoxins (*e.g.*, nodularin and microcystin) that can severely affect human health [10]. Particularly, recent reports have indicated that β-N-methylamino-l-alanine (BMAA), a neurotoxic chemical found in cyanobacteria can have long-standing and serious health effects [11]. However, despite producing these toxins, some cyanobacteria might be suitable for biodiesel production since of their high lipid yields, thereby compensating for their toxicity. Biodiesels can thus be derived from cyanobacterial fatty acids followed by transesterification to obtain high-purity, biodegradable, and non-toxic fuels [12].

The utmost importance in the biodiesel process is the initial selection of cyanobacterial strains. The bioprospecting and isolation of natural endogenous microalgae enables the development of species-specific production of viable chemicals and biodiesels [13]. Particularly, native isolated strains have significant commercial value for regional large-scale production, owing to their robust growth under conditions to which they are naturally adapted [14]. The approach also prevents unexpected ecological risks from the potential introduction of invasive species in commercial cultivation fields. In this study, we isolated and identified a native cyanobacterium belonging to the genus *Pseudanabaena* in South Korea and improved the biomass productivity by optimizing the cultivation conditions. Additionally, a focus on estimating the characteristics of biodiesel obtained from cyanobacterium was conducted, as all biodiesel properties must be considered to meet standard requirements but are often overlooked in many studies for biodiesel production. Here, we report the properties and potential large-scale production of biodiesel derived from a newly isolated natural strain of *Pseudanabaena* from a quantitative and qualitative perspective.

## Materials and Methods

### Isolation, Purification, and Phylogenic Identification

Field collections were conducted near the Gangjeong-Goryeong weir of the Nakdong River (35°51'31.6"N 128°23'23.0"E) in May and June of 2020. The 16S rRNA gene sequence of strain GO0704 was compared with those of 15 *Pseudanabaena* species from the NCBI database. The sequences were aligned using Clustal W in the MEGA-X program (Molecular Evolutionary Genetics Analysis across computing platforms). The outgroup was hypothesized as *Gloeobacter violaceus* PCC 7421. Pairwise distances were calculated using the maximum composite likelihood model. Datasets were analyzed using maximum likelihood analysis with 1,000 bootstrap replicates for testing the robustness of each clade using the MEGA-X program.

### Maintenance and Cultivation Conditions

*Pseudanabaena mucicola* GO0704 was pre-cultivated in a 500 ml Erlenmeyer flask with 300 m of aseptic BG-11 medium. The BG-11 medium was prepared as described in a previous research [15]. The cells were maintained with aeration at a 90 μmol photons/m^2^/s light intensity and a temperature of 23°C. Pre-culture was performed at 10-day intervals to maintain the stock in good condition, after which the stock culture was used at the start of the experiment.

### Culture of the Cyanobacteria

Batch cultures were performed in both flask and photobioreactor under sterilized conditions. First, the effects of different organic carbon sources on mixotrophic cultures of *P. mucicola* GO0074 were tested in a 250 ml flask. The BG-11 growth medium was supplemented with either glucose, galactose, xylose, or sodium acetate at an equivalent carbon content (1 g/l). Additionally, to confirm the dose-dependent effect of sodium acetate on *P. mucicola* GO0704, different concentrations of sodium acetate (1, 5, and 10 g/l) were added to the BG-11 medium. As a control, a nontreated BG-11 medium was prepared, and all media were autoclaved at 121°C for 20 min. The pre-cultured stocks were then inoculated (5% (v/v)) into 250 ml Erlenmeyer flasks containing 200 ml of prepared mediums. Flask experiments were conducted under a light intensity of 130 μmol photons/m^2^/s and 200 RPM of shaking at 25°C for 120 h.

Based on the outcomes of the flask experiments, the native cyanobacteria were cultivated in a 5 L stirred glass bioreactor (CNS Co. Ltd., Korea). The initial volume of BG-11 medium was 3 L, and only the treatment group was supplemented with 1 g/L of sodium acetate. The inoculation concentration was 5% (v/v) of precultured cells, as in the flask experiments. The initial pH of the culture was set to 7.5 using 1 N NaOH/HCl. The cultures were continuously aerated (1 ml/min) and agitated (300 RPM) under LED light at 25 °C. The LED lights were installed on both sides of the bioreactor to provide a constant light intensity (100–120 μmol photons/m^2^/s) into the photobioreactor.

### Measurement of Cell Growth

Cell growth was monitored based on wavelength of 650 nm (OD_650nm_), using a Genesys 10S UV-Vis spectrophotometer (Thermo Scientific). Cell numbers were estimated based on the value of OD_650nm_. Parallelly, to measure dry weight, 5 ml of cyanobacterial biomass was harvested by centrifugation (3,500 rpm for 10 min) and filtered with 1.2 μm glass fiber filters (USA) followed by weighing after thoroughly drying the samples in an oven.

### Analysis of Chlorophyll-a, Phycobiliproteins, and Fatty Acids

Chlorophyll-a contents were analyzed using a Genesys 10S UV-Vis spectrophotometer (Thermo Scientific). To extract Chlorophyll-a from cyanobacterial biomass, 1ml of cell suspensions were centrifuged, and the supernatant was removed. The cells were then mixed with 1 ml of 95% ethanol and incubated in the dark for 24 h at 4°C. The mixture was centrifuged at 8,000 rpm for 5 min to remove cell debris and then the absorbance of the supernatant liquid was measured at 665 and 652 nm by spectrophotometry. The concentration of chlorophyll-a was calculated according to [16].

Chlorophyll-a (μg/ml) = 8.096 (OD_652nm_) + 16.52 (OD_665nm_)

To extract phycobiliproteins, dried cyanobacterial biomass (40 mg) was vortexed with 10 ml of distilled water. After freezing the samples at −80°C for 2 h, the cyanobacterial biomass was incubated at 4°C overnight. Next, the samples were centrifuged at 3,500 ×*g* for 10 min. Supernatants were collected, and absorbance was read at 562, 615, and 652 nm using a 96-well plate. Phycocyanin (PC), phycoerythrin (PE), and allophycocyanin (APC) concentrations were calculated according to a previous report [17].

Phycocyanin (PC) (mg/ml) = [OD_620nm_ – 0.474(OD_652nm_)] / 5.34

Allophycocyanin (APC) (mg/ml) = [OD_652nm_ – 0.208(OD620nm) / 5.09

Phycoerythrin (PE) (mg/ml) = [OD_562nm_ – 2.41(PC) – 0.849 (APC) / 9.62

Extractions of fatty acids and transesterification were sequentially conducted using fatty acid methyl ester (FAME) conversion kits (a fatty acid methylation kit and a FAME purification kit) (Sigma-Aldrich, USA) using 10 mg of a freeze-dried cyanobacterial sample. The refined FAME content and composition were analyzed with a gas chromatography-mass spectrometer (GC-MS) (Agilent 6890N Network GC system, 5975 inert mass selective detector) equipped with an HP-5MS column (length: 30 m, inner diameter: 0.25 mm, film thickness: 0.2 μm)(Agilent Technologies, USA). Gas chromatography-mass spectrometry (GC-MS) was conducted under the following conditions: carrier gas (He) flow rate, 1mL/min; the oven temperature was initially held at 70°C for a 1-min isothermal period, heated to 76°C at a 1°C/min rate, then to 350°C at a 6°C/min rate and held for 1 min. The Supelco 37 Component FAME mixture (Sigma-Aldrich) was provided as an external standard.

## Results and Discussion

### Morphology and Phylogenetic Analysis

**Fig. 1:**
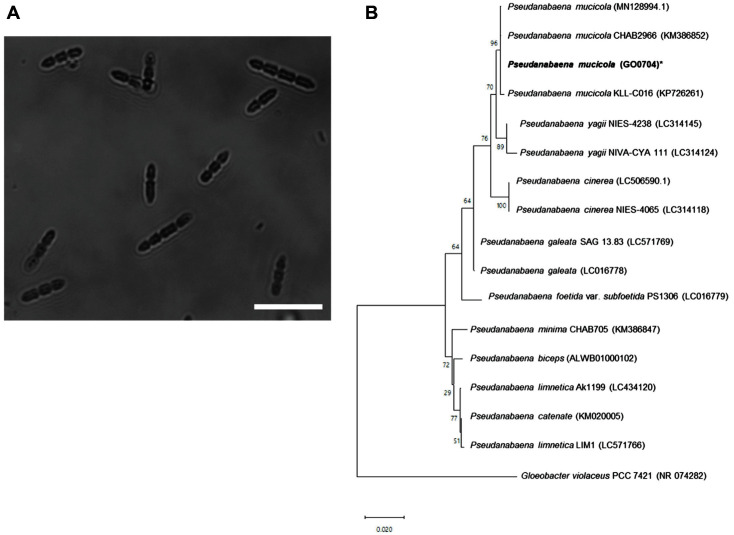
Morphology and phylogenetic analysis of isolated cyanobacterium. (**A**) Microscope images of isolated strain from environmental sample (scale bar: 20 μm). (**B**) Phylogenetic tree with the isolated strain. The number in bracket indicate the GenBank accession number.

A cyanobacterial strain was collected from the Gangjeong-Goryeong weir of the Nakdong River in Goryeong, Republic of Korea. Morphological characterization of the strain was conducted under a bright-field microscope (Fig. 1A). The native cyanobacterial cells isolated in this study were filamentous and arranged in lines of two to six 3–20 μm cells. The morphology of the cells was largely consistent with that of *Pseudananbaena* sp.

To confirm our morphological analyses, molecular identification was performed and a phylogenetic tree was constructed based on the results of molecular phylogenetic analysis of 16s rRNA sequences (Fig. 1B). As expected, a total of 1,484 bp of the 16S rRNA gene sequences were aligned with 16 members of the *Pseudanabaena* genus. Two Korean populations of *P. mucicola* (GO0704 isolated in this study, and MN128994) were identical to each other. Moreover, these strains shared high similarity with a Chinese population (KM386852) of *P. mucicola*, with only a 0.04% dissimilarity. *P. mucicola* GO0704 was also closely related to other intrageneric species such as *P. yagii* (0.5-0.8%), *P. galeata* (0.9%), and *P. cinerea* (1%).

### Effect of Sodium Acetate on Native *Pseudanabaena mucicola* GO0704 Growth

Due to the specific shape of Pseudananbaena sp., previous studies on Pseudananbaena sp. mainly used OD value as an indirect indicator of cell growth [17, 18]. Here, we also confirmed the strong correlation between OD_650nm_ and cell number (coefficients R^2^ = 0.9976) (Fig. S1).

**Fig. 2:**
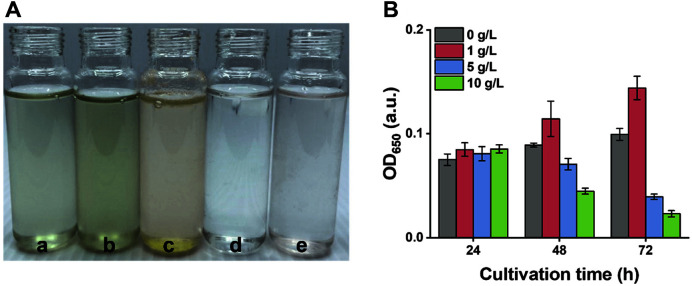
Effect of different organic carbon sources in mixotrophic cultivation of *Pseudanabaena mucicola* GO0704. (**A**) Screening of optimal carbon sources (a: control, b: sodium acetate, c: glucose, d: galactose, e: xylose). (**B**) Growth curve according to sodium acetate concentrations (0, 1, 5, and 10 g/l).

Cyanobacteria are capable of growing in three trophic (photo-/mixo-/hetero-) modes [5]. The mixotrophic mode uses both organic carbon and CO_2_ in the energy cycle [19]. In this mode, two carbon metabolic ways act in conjunction. Therefore, organic carbon can be used as an energy source in addition to radiant energy [20]. Next, we identified the most effective carbon source for the growth of *P. mucicola* GO0704. Equal amounts (1 g/l) of different carbon sources were supplied as nutrients for *P. mucicola* GO0704: glucose, xylose, galactose, and sodium acetate (Fig. 2A). After 72 h of cultivation, sodium acetate was the only carbon source that stimulated the growth of *P. mucicola* GO0704. Previous studies have demonstrated that sodium acetate can enhance the production of cyanobacterial biomass and metabolites [21]. In contrast, the addition of galactose and xylose resulted in the death of the cyanobacterial cells, indicating that these carbohydrates adversely affect the cells. Specifically, a previous study reported that xylose induced the death of microalgal cell by blocking carbon cycle of photosynthetic system as a competitive inhibitor [22]. These results suggest that sodium acetate is an appropriate organic carbon source for mixotrophic cultivation of the cyanobacterium *P. mucicola*.

We also investigated the optimal initial sodium acetate concentration (0, 1, 5, and 10 g/l) for maximizing biomass production. As shown in Fig. 2B, the 1 g/l sodium acetate concentration showed an obvious increase in the growth of *P. mucicola* GO0704, reaching approximately 45% in 72 h. In contrast, at concentrations above 1 g/l, as with the higher concentration of sodium acetate, *P. mucicola* growth became inhibited. The OD650 values of the 5 g/l and 10 g/l treatment of sodium acetate were significantly decreased to 0.039 and 0.023, respectively, at 72 h.

This inhibition might be caused by damage to the oxygen-evolving complex at the donor side of photosystem II (PSII) by a high concentration of sodium acetate [23]. PSII is a major protein complex involved in the photosynthesis process and catalyzes the light-induced release of oxygen and the reduction of plastoquinone in water [24]. Therefore, inhibition of PSII evidently reduces photosynthesis, which is the basic survival strategy of cyanobacteria. Based on the above results, the ideal sodium acetate concentration was 1 g/l.

### Production of Value-Added Chemicals from *Pseudanabaena mucicola* GO0704

**Fig. 3:**
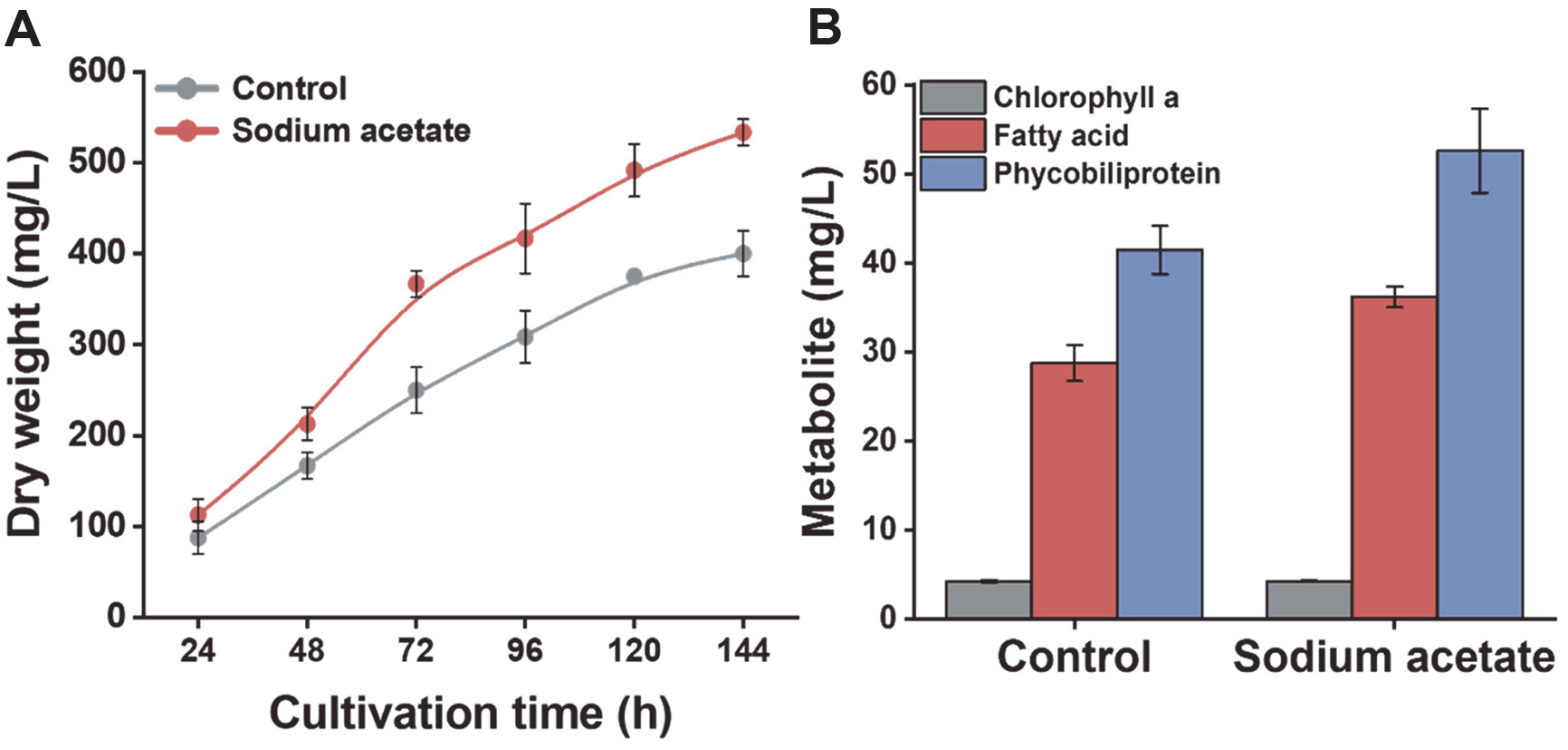
Effect of sodium acetate treatment on dry weight and metabolites of *Pseudanabaena mucicola* GO0704 in a 5 L stirred-tank bioreactor. (**A**) Dry weight of *P. mucicola* GO0704. (**B**) Production of chlorophyll-a, fatty acid, and phycobiliprotein.

Next, a scale-up study in batch bioreactor was conducted to examine the effect of sodium acetate addition on metabolite production and its industrial applicability. Fig. 3A illustrates the effect of sodium acetate treatment on biomass production in a 5 L stirred-tank bioreactor. At 144 h, the dry weight of cells supplemented with sodium acetate increased 1.3-fold compared with the control (sodium acetate: 530 mg/l, control: 400 mg/l). Sodium acetate treatment in the photobioreactor promoted the rapid growth of cells, similar to the flask experiment results. The gradual scale-up of bioreactors has recently garnered increasing attention, as this constitutes essential research to assess the feasibility of industrial-scale commercialization. This is because cultivation in flasks and bioreactors can result in different outcomes due to differences in scale and environmental factors such as aeration rate, agitation rate, and shear stress [25, 26]. Therefore, we believe that this research is important to evaluate the potential industrial applicability of the mixotrophic mode of *P. mucicola*.

Next, we explored the production of diverse metabolites by *P. mucicola* GO0704 in a 5 L stirred-tank bioreactor. As illustrated in Fig. 3B, the productivity of value-added metabolites increased with biomass productivity. *Pseudanabaena* sp. is generally known to produce useful substances such as chlorophyll, phycobiliproteins, and lipids [17, 18]. Chlorophyll-a is the most abundant green pigment in cyanobacteria playing a central role in photosynthesis [27]. It is a commercially important natural dye and is used to color inks, cosmetics, perfumes, liniments, and leather [27]. Our results showed similar chlorophyll-a productions of 4.26 mg/l and 4.27 mg/l in the control and sodium acetate supplementation groups. Unlike the enhanced biomass productivity in the treatment group, there was no change in the productivity of chlorophyll-a. Sodium acetate treatment appears to reduce chlorophyll a content. chlorophyll a content is known to vary according to the cultivation conditions (*e.g.*, type and intensity of light, nutrient composition, and temperature) [28]. Consumption of organic carbon sources by photosynthetic microorganisms in mixotrophic cultivation can lead to a decrease in chlorophyll content due to changes in photosystem activity [29]. Previous reports have shown that organic carbon sources cause a decrease in the amount of excitation energy retained in PSII, resulting in a decrease in photosystem II (PSII) activity [30]. Photosystem II activity represents a photosynthetic efficiency, and thus acts as a factor that can indirectly prove the decrease in the content of chlorophyll a.

Phycobiliprotein is a pigment-protein complex responsible for light collection in cyanobacteria and is a value-added substance with diverse potential uses in the cosmetic, food, health, and medical industries [31]. Phycobiliprotein is composed of phycocyanin (620 nm), allophycocyanin (652 nm), and phycoerythrin (565 nm), which can be distinguished based on the light absorption wavelength [31]. The phycobiliprotein concentration in the *P. mucicola* GO0704 cell culture was 52.58 mg/l, which accounted for a 27% increase in mixotrophic conditions. According to (Table 1), most of the phycobiliprotein observed in our study was phycocyanin (PC), and the acetate-treated group produced 52.58 mg/l and the control group produced 31.39 mg/l of PC. There were no significant differences in phycocyanin content between the acetate supplemented group and the control group. Previous research has reported that the accumulation of PC in diverse species was promoted in the mixotrophic condition [32-34]. This discrepancy might be due to the result of a complex PC accumulation regulation mechanism caused by the difference in the types of carbon sources [35]. it was found that *G. sulphuraria* grown on glycerol under certain light conditions stimulated the accumulation of PC, but not those grown on glucose and fructose [36, 37]. Treatment of glucose, lactose, and galactose also showed an increase in PC content of *Anabaena variabilis*, whereas treatment of fructose and sucrose showed no significant change in it [38]. However, when combined with biomass productivity had led to higher PC productivity in the treated group. Among phycobiliprotein, phycocyanin is the most widely studied pigment and has been the focus of many natural bioactive compound screening studies [31]. Phycocyanin has been reported to possess antioxidant, anti-inflammatory, and immune-stimulating properties [31]. Moreover, It has been reported to block the cell cycle and could thus be used as an effective anticancer agent [39]. Our results suggested that the *P. mucicola* strain GO0704 could enhance the productivity of phycocyanin by acetate treatment and further potentially be used as a natural source of bioactive substances that enhance human health.

However, prior to the commercialization of food and biomedical products, candidate compounds must pass a rigorous safety examination. In contrast, biodiesel applications of cyanobacteria such as *P. mucicola* GO0704 are relatively unaffected by the toxicity of cyanobacteria. Therefore, the utilization of cyanobacteria cells as a biodiesel source is not limited by the safety requirements of products intended to be used in the food and medical industries. Fatty acids are the main components of biodiesel, and the total fatty acid production of *P. mucicola* GO0704 was 36.17 mg/l. The maximum yield was achieved in the sodium acetate treatment group at 144 h, which constituted a 26% increase compared to the control group (Fig. 3B). In the sodium acetate treatment, the fatty acid content varied with the cultivation period. The content of total fatty acids reached 5.8%, 8.1%, and 6.8% at 48 h, 96 h, and 144 h, respectively (*i.e.*, the highest fatty acid content was achieved at 96 h) (Fig. S2B). The decrease in fatty acid content observed at 144 h might be related to the complete consumption of sodium acetate, as sodium acetate is often used as a carbon source to enhance total lipid and fatty acid contents [40, 41]. Notably, sodium acetate addition at the initial culture stage induced fatty acid accumulation. After all carbon sources were consumed within 144 h, the mixotrophic mode might have gradually shifted to phototrophic growth. The decrease in fatty acids can be attributed to the fact that accumulated metabolites such as fatty acids are often used as energy sources for cell growth. Given that the fatty acids content of *P. mucicola* GO0704 was reduced after depletion of sodium acetate, additional supplementation of carbon sources could further elevate the maximum contents of fatty acids, which leaves for further research.

Biodiesel productivity is a critical variable in selecting the most appropriate species for biodiesel production [42]. This factor is also a practical indicator for verifying the economic feasibility of biodiesel commercialization [43]. Biodiesel productivity is calculated as fatty acid content and biomass production divided by the culture time required to achieve a profitable cell density. In this study, the maximum biodiesel productivity rates at 48, 96, and 144 h were 6.2, 8.1, and 6.3 mg/l/d in mixotrophic conditions. Therefore, the *P. mucicola* GO0704 cultures should ideally be harvested at 96 h to maximize biodiesel yields. Additionally, the biodiesel productivity of most isolated cyanobacteria is lower than that of *P. mucicola* GO0704 (8.1 mg/l/d), thus highlighting its applicability as a bioenergy source (Table 2).

### Fatty Acid Profiling in Two Trophic (photo-/mixo-) Culture Modes

Next, detailed fatty acid compositions were studied through conventional FAME extraction and conversion procedures, and a total of 8 fatty acids were detected, including 4 saturated fatty acids (SFAs), 2 monounsaturated fatty acids (MUFAs), and 2 polyunsaturated fatty acids (PUFAs). In the case of supplementation with sodium acetate, the percentage of C12:0, C16:0, C16:1, C18:0, and C18:1 increased, whereas the contents of C14:0, C18:2, and C18:3 decreased (Table 3). Along with the decrease in the percentage of total PUFA, the levels of total MUFAs increased. Consistent with these observations, the conversion of PUFAs to MUFAs under mixotrophic conditions has been frequently reported in previous studies [44-46]. The C/N ratio of the medium is known to have a significant influence on the fatty acid composition of microalgae [47]. Likewise, it can be inferred that PUFAs were more readily converted to MUFAs under mixotrophic conditions in cyanobacteria such as *P. mucicola* GO0704.

Increases in the proportion of MUFAs such as C18:1 improves the ignition performance of biodiesel by effectively negating the poor oxidation stability of PUFAs [48]. However, the composition of SFAs is also known to have a crucial impact on the low-temperature fluidity of biodiesel [49]. Therefore, ideal biodiesel production is highly complex, thus requiring a balance of properties such as ignition efficiency, low-temperature fluidity, and oxidation stability. Therefore, in-depth fatty acid composition analyses must be conducted to select optimal biodiesel sources. To accurately evaluate the quality of FAMEs in biodiesel, the expected properties must be carefully cross-checked with existing references.

### Quality Assessment of Biodiesel Derived from *Pseudanabaena mucicola* GO0704

The quality of FAMEs derived from *P. mucicola* GO0704 was predicted using the Biodiesel Analyzer software [50]. The properties of biodiesels derived from *P. mucicola* GO0704 and other microalgae (*Euglena*), soybean biodiesel (currently the main source of biodiesel), and international biodiesel standards [ASTM 6761 (USA) and EN 14214 (Europe)] were carefully compared to assess the quality of *P. mucicola* GO0704-derived biodiesel (Table 4). To this end, important properties often reported in the other studies for the evaluation of biodiesel were considered, including viscosity, cloud point, low-temperature filter plugging point, pour point, cetane number, and iodine value.

First, the cetane number of biodiesels derived from *P. mucicola* was 56.2, which is higher than that of soybean (49.0). In general, a higher cetane number suggests a shorter infusion delay [51]. *P. mucicola* biodiesel, which exhibited a higher cetane number than that of soybean, may thus not be affected by delayed infusion. Biodiesel derived from *P. mucicola* also has a lower viscosity than those of soybean oil and Euglena. High viscosity causes fogging problems and may form engine deposits [52]. The iodine value (gI_2_ 100/g) measured in *P. mucicola*-based biodiesel (65.1) was approximately 0.5 times that of soybean oil (128) and lower than that of Euglena-derived biodiesel (118). The iodine value is an important index of the degree of unsaturation contained in fatty acids and a decrease in unsaturation leads to an increase in oxidative stability [53]. The cloud point of *P. mucicola* is 3.9°C, which is higher than the −3°C of the ASTM 6761 (US) standard specification. Diesel fuel crystallizes with apparent solidification and turns turbid at a temperature known as the cloud point. A high cloud point causes clogging of fuel lines and filters due to crystallization [52]. *Euglena* biodiesel with a CP point of 15°C poses a greater risk of filter clogging in colder regions. Soybeans with high cold filter plugging point also have the same issue, which could be only possibly reverted with the incorporation of additives into fuels. For example, a nearly 30% decrease in the fuel pour point temperature was achieved when using a 20% ethanol fuel mixture [54]. Taken together, our findings indicated that the native cyanobacterial strain (*P. mucicola* Go0704) isolated and characterized in this study met most of the standard properties of ASTM6761 (USA) and EN 14214 (Europe), and the potential problem of CP could be solved using additives. *P. mucicola*-derived biodiesel displayed better performance than soybean oil, the first-generation biodiesel, and Euglena-based biodiesel, a representative microalga-derived biodiesel. In summary, the native strain *P. mucicola* GO0704 is worth considering as a potential biodiesel resource. However, a comprehensive analysis of *P. mucicola* GO0704-derived biodiesel production from cultivation to conversion is necessary to confirm its feasibility.

### Feasibility of Mixotrophic Cultivation of *Pseudanabaena mucicola* GO0704 Using Acetate Obtained from Organic Wastes

Acetate can be readily obtained from the acetic acid fermentation process of organic wastes like wastewater and food waste [55]. Table 5 shows the conversion of organic waste to acetate by anaerobic processes. Food waste (96.0 g COD/L) can be converted into acetate (25.9 g/l), reaching a maximum conversion yield of 27% [56]. Massive amounts of food waste are generated each year (1.3 billion tons) [57] and therefore acetate could be obtained from food waste for mixotrophic cultivation. Moreover, given that the cost of organic carbon sources is among the highest expenses in microalgae cultivation [58], the utilization of acetate obtained from food waste offers a great opportunity to reduce not only the disposal of food waste but also the cost of biodiesel production from cyanobacteria.

Our study comprehensively assessed the entire cyanobacteria-derived biodiesel production process, from the isolation and identification of the cyanobacteria strain, the application of the mixotrophic mode in large-scale cultivation, and the analysis of valuable substances with an emphasis on biodiesel performance prediction. The cyanobacteria collected from Goryeong, Republic of Korea, was identified as *P. mucicola* (strain name, GO0704). Sodium acetate has been shown to induce the mixotrophic mode and enhance the productivity of phycobiliprotein and fatty acids in large-scale cultivation. Sodium acetate treatment improved the quality of biodiesel by enhancing the fatty acid composition. The potential of *P. mucicola* GO0704 for biodiesel production was also carefully evaluated by analyzing the biodiesel properties and comparing them to international standards. Our study is the first to demonstrate the potential of an indigenous strain of *P. mucicola* for biodiesel in large-scale mixotrophic cultures and provides a basis for the future development of *P. mucicola*-derived biodiesel production.

## Figures and Tables

**Table 1:** Compositional profile of phycobiliprotein of *Pseudanabaena mucicola* GO0704.

Pigment	Control group		Sodium acetate treatment group	
	
	Pigment content (mg/g)	Pigment productivity (mg/l)	Pigment content (mg/g)	Pigment productivity (mg/l)
Phycocyanin	78.49	31.39	76.98	40.80
Allophycocyanin	24.62	9.84	21.85	11.58
Phycoerythrin	0.40	0.16	0.38	0.20

**Total Phycobiliprotein**	**103.51**	**41.40**	**99.21**	**52.58**

**Table 2:** Comparison of biodiesel productivity of *Pseudanabaena mucicola* GO0704 with 13 previously characterized cyanobacterial strains.

Species	Cultivation time (days)	Dry weight (g/l)	Biomass productivity (mg/l/d)	Biodiesel productivity (mg/l/d)	Reference
*Oscillatoria* sp. FW01	26	1.8	70.4	8.8	[59]
*Calothrix* sp. MBDU 013	24	0.5	20.7	2.4	[60]
*Anabaena sphaerica* MBDU 105	24	0.2	9.33	1.7	[60]
*Nostoc* sp. MBDU 013	24	0.0	2	1.4	[60]
*Synechococcus* sp.	13	0.4	21	2.6	[61]
*Synechocystis* sp. PCC 6803	19	1.2	62.6	8.2	[62]
Nostoc muscorum	19	0.8	40.5	3.0	[62]
*Synechococcus* sp. PCC 7942	19	1.1	57.4	6.3	[62]
*Oscillatoria* sp.	19	0.8	40.0	3.4	[62]
*Anabaena cylindrica*	19	0.8	42.6	2.0	[62]
*Lyngbya* sp.	19	0.6	33.7	3.5	[62]
*Phormidium* sp.	19	1.1	56.3	4.7	[62]
*Pseudanabaena* sp. CY14-1	6	0.4	73.3	8.1	[18]
*Pseudanabaena mucicola* GO0704	4	0.4	100.0	8.1	This study

**Table 3:** Comparison of FAME content between 1 g/l sodium acetate treated cells and the control.

Fatty Acid	Control group		Sodium acetate treatment group	
	
	Fatty acid composition (%)	Fatty acid productivity (mg/l)	Fatty acid composition (%)	Fatty acid productivity (mg/l)
C12:0	5.47	1.19	7.00	2.37
C14:0	31.78	6.94	28.45	9.64
C16:0	14.85	3.25	16.38	5.55
C16:1	6.38	1.39	6.72	2.28
C18:0	0.87	0.19	1.88	0.64
C18:1	17.26	3.80	19.34	6.56
C18:2	18.45	3.77	15.59	5.29
C18:3	4.94	1.08	4.68	1.57

SFA	52.97	11.57	53.71	18.21
MUFA	23.65	5.17	26.06	8.83
PUFA	23.39	5.11	20.23	6.86

**TFA**	**100**	**21.85**	**100**	**33.90**

*SFAs: saturated fatty acids; MUFAs: monounsaturated fatty acids; PUFAs: polyunsaturated fatty acids; TFA: total fatty acids.

**Table 4:** Biodiesel properties of *Pseudanabaena mucicola* GO0704 compared with Euglena and three standards.

Properties	*Pseudanabaena mucicola* GO0704	Euglena [63]	Soybean oil [64]	EN 14214 [64]	ASTM D6751 [64]
CN	56.2	65	49.0	Min. 51	Min. 47
IV (gI_2_ 100/g)	65.1	118	128	Max. 120	-
DU (%)	66.6	-	143.8	-	-
SV	222.5	-	-	-	-
LCSF (%)	2.6	-	1.6	-	-
CFPP (°C)	-8.4	-	-5.0	-	-
CP (°C)	3.6	15	-	-	-3 to -12
OS (h)	8.4	6.2	1.3	Min. 3	Min. 6
μ (mm^2^/s)	3.3	4.525	4.2	3.5 - 5.0	1.9 - 6.0
ρ (g/cm^3^)	0.875	0.868	-	-	-

*CN: cetane number; IV: iodine value; DU: degree of unsaturation; SV: saponification value; LCSF: long-chain saturated factor; CFPP: cold filter plugging point; CP: cloud point; OS: oxidation stability; μ: kinematic viscosity; ρ: kinematic density.

**Table 5:** Production of acetate from waste feedstocks.

Feedstock	Inoculum	Acetate (g/l)	Reference
Food waste (96.0 g COD/L)	Yeast, acetic acid bacteria	25.9	[56]
Food waste (15.0 g COD/L)	Anaerobic sludge	4.0	[65]
Sewage sludge (1.0 g COD/L)	Anaerobic sludge	0.3	[66]
Waste activated sludge (16.2 g VS/L)	Anaerobic sludge	2.1	[67]
Waste mushroom	*Acetobacter pasteurianus*	38.0	[68]
Food waste (49.3 g VS/L)	Mesophilic digested sludge	18.4	[69]

*COD: chemical oxygen demand; VS: volatile solids.
